# High sustained efficacy of a prophylactic quadrivalent human papillomavirus types 6/11/16/18 L1 virus-like particle vaccine through 5 years of follow-up

**DOI:** 10.1038/sj.bjc.6603469

**Published:** 2006-11-21

**Authors:** L L Villa, R L R Costa, C A Petta, R P Andrade, J Paavonen, O-E Iversen, S-E Olsson, J Høye, M Steinwall, G Riis-Johannessen, A Andersson-Ellstrom, K Elfgren, G von Krogh, M Lehtinen, C Malm, G M Tamms, K Giacoletti, L Lupinacci, R Railkar, F J Taddeo, J Bryan, M T Esser, H L Sings, A J Saah, E Barr

**Affiliations:** 1 Ludwig Institute for Cancer Research, R Prof Antonio Prudente 109, 01509-010 Sao Paulo, SP, Brazil; 2 Department of Gynecology, Instituto Brasileiro de Controle do Cancer, and Hospital do Cancer, R Prof Antonio Prudente 109, 01509-010 Sao Paulo, Brazil; 3 Department of Obstetrics and Gynecology, Universidade Estadual de Campinas, Rua Eduardo Lane 380, 13073-002 Campinas, Sao Paulo, Brazil; 4 CERHFAC – Center of Studies and Clinical Research, Rua Amâncio Moro, 77–Alto da Glória 80030-220, Curitiba-Paraná-Brazil; 5 Department of Obstetrics and Gynecology, University of Helsinki, 00290 Helsinki, Finland; 6 Womens Clinic, Haukeland University Hospital, University of Bergen, N-5021 Bergen, Norway; 7 Karolinska Institute at Danderyds Hospital, SE-182 88 Danderyd, Sweden; 8 Nedre Storgate 31, 3015 Drammen, Norway; 9 Department of Obstetrics and Gynecology, University of Lund, Malmö University Hospital, S-205 02 Malmö, Sweden; 10 Gynekologene på Kolbotn, Postboks 183, 1411 Kolbotn, Norway; 11 Department of Obstetrics and Gynecology, Sahlgrenska Academy at Göteborgs University, S 416 85 Göteborg, Sweden; 12 CLINTEC, Department of Obstetrics and Gynecology, Karolinska University Hospital Huddinge, 141 86 Stockholm, Sweden; 13 Department of Dermatovenereology, Karolinska University Hospital Solna, 171 76 Stockholm, Sweden; 14 Department of Infectious Disease Epidemiology, National Public Health Institute, KTL, Aapistie 1a, 90520 Oulu, Finland; 15 Department of Student Health Service, University of Helsinki, Tööiönkatu 37A, 00260 Helsinki, Finland; 16 Department of Vaccines and Biologics Clinical Research, Merck Research Laboratories, 770 Sumneytown Pike, West Point, PA 19486, USA; 17 Department of Biostatistics, Merck Research Laboratories, 770 Sumneytown Pike, West Point, PA 19486, USA; 18 Department of Vaccine and Biologics Research, Merck Research Laboratories, 770 Sumneytown Pike, West Point, PA 19486, USA; 19 Department of Medical Communications, Merck Research Laboratories, 770 Sumneytown Pike, West Point, PA 19486, USA

**Keywords:** human papillomavirus, prophylactic vaccine, virus-like particles, cervical intraepithelial neoplasia (CIN), genital warts, cervical cancer, clinical trial

## Abstract

Human papillomavirus (HPV) causes cervical, vulvar, and vaginal cancers, precancerous dysplasia, and genital warts. We report data for the longest efficacy evaluation to date of a prophylactic HPV vaccine. In total, 552 women (16–23 years) were enrolled in a randomised, placebo-controlled study of a quadrivalent HPV 6/11/16/18 L1 virus-like-particle vaccine with vaccination at months 0, 2, and 6. At regular intervals through 3 years, subjects underwent gynaecologic examination, cervicovaginal sampling for HPV DNA, serum anti-HPV testing, and Pap testing, with follow-up biopsy as indicated. A subset of 241 subjects underwent two further years of follow-up. At 5 years post enrolment, the combined incidence of HPV 6/11/16/18-related persistent infection or disease was reduced in vaccine-recipients by 96% (two cases vaccine versus 46 placebo). There were no cases of HPV 6/11/16/18-related precancerous cervical dysplasia or genital warts in vaccine recipients, and six cases in placebo recipients (efficacy=100%; 95% CI:12–100%). Through 5 years, vaccine-induced anti-HPV geometric mean titres remained at or above those following natural infection. In conclusion, a prophylactic quadrivalent HPV vaccine was effective through 5 years for prevention of persistent infection and disease caused by HPV 6/11/16/18. This duration supports vaccination of adolescents and young adults, which is expected to greatly reduce the burden of cervical and genital cancers, precancerous dysplasia, and genital warts.

The human papillomavirus (HPV) family is a heterogeneous group of epitheliotropic viruses of which more than 100 genotypes have been fully sequenced to date (de Villiers *et al*, 2004). The primary target of HPV infection is the basal cell of the squamous epithelium. In men and women, anogenital infection with HPV can manifest itself as any one of a large number of subclinical and clinical manifestations including anogenital neoplasia, cancer, and genital warts (Gissmann and zur Hausen, 1980; Jones *et al*, 1997; Goodman, 1998; Bosch *et al*, 2002; Partridge and Koutsky, 2006).

HPV infection is common, with a lifetime risk exceeding 50% (World Health Organization (WHO, 2004)). The prevalence of HPV in the general population is estimated between 9 and 13% worldwide and varies between 1.6 and 25.6% depending on the country (World Health Organization, 2004; Clifford *et al*, 2005). A significant proportion of HPV disease is attributed to four types: 6, 11, 16 and 18. HPV types 16 and 18 cause approximately 70% of all cervical cancer cases worldwide (Muñoz *et al*, 2003) and a significant proportion of vaginal (Daling *et al*, 2002), vulvar (Madeleine *et al*, 1997), anal (Frisch, 2002) penile (Partridge and Koutsky, 2006), and head and neck cancers (Gillison and Lowy, 2006). HPV 6 and 11 infections in men and women are responsible for the great majority of genital wart cases (Wiley *et al*, 2002) and recurrent respiratory papillomatosis, an aggressive, highly morbid and occasionally fatal disease manifested as rapidly growing benign laryngeal tumours that cause airway obstruction (Somers *et al*, 1997; Derkay and Darrow, 2000; Green *et al*, 2000).

In a recent multinational study conducted in 5455 young women who reported a lifetime history of four or fewer sexual partners, nearly one-third had serologic or molecular evidence of infection with high-risk (oncogenic) HPV types 16 or 18, or low-risk (nononcogenic) HPV types 6 or 11 (Sattler, for the FUTURE I Investigators, 2005). Owing to the high observed prevalence of HPV 6, 11, 16, and 18 and their causative association with anogenital cancers, dysplasia or genital warts, a prophylactic vaccine covering these types has the potential to substantially reduce the burden of clinical HPV disease in both men and women. Phase III trials conducted in >18 000 women have demonstrated that a three-dose regimen of quadrivalent HPV 6/11/16/18 L1 virus-like particle (VLP) vaccine was 100% effective in preventing obligate precancerous lesions of the cervix, vagina, and vulva in women who were naïve to HPV 6, 11, 16, or 18 through completion of the vaccination regimen (Sattler, for the FUTURE I Investigators, 2005). In addition, prophylactic administration of a three-dose regimen of the vaccine was 100% effective against genital warts (Sattler, for the FUTURE I Investigators, 2005). In these Phase III trials, women were followed for an average of 2 years postvaccination. As the risk of HPV infection is life long, HPV vaccines must induce long-term protection. Here, we report quadrivalent vaccine efficacy (VE) and immunogenicity through 5 years following vaccination, representing the longest term evaluation to date of an HPV vaccine.

## MATERIALS AND METHODS

### Study design

The trial (Merck protocol V501-007) was a Phase II, randomised, multicentre, double-blind, placebo-controlled study of a quadrivalent HPV 6/11/16/18 L1 VLP vaccine. A total of 1158 women aged 16–23 years were recruited in Brazil, Nordic countries (Finland, Sweden, Norway), and the USA. The study enrolled nonpregnant, healthy women who had no prior abnormal Pap smears, and reported a lifetime history of four or fewer male sex partners. Among virgins, enrolment was limited to those women who were ⩾18 years of age and seeking contraception. This study did not exclude subjects with prior HPV infection. Participants were asked to use effective contraception during the trial. All subjects or parents/legal guardians signed informed consents following review of the protocol procedures. The study was conducted in conformance with applicable country or local requirements regarding ethical committee review, informed consent, and other statutes or regulations regarding the protection of the rights and welfare of human subjects participating in biomedical research.

The study evaluated three formulations of a quadrivalent HPV 6/11/16/18 L1 VLP vaccine. The study included two placebo arms with different adjuvant doses (225 or 450 *μ*g) to provide appropriate safety comparators for the different vaccine formulations. Efficacy, safety, and tolerability through 3 years have been described for the three formulations (Villa *et al*, 2005; Villa *et al*, 2006). Of the 551 women who were randomised and vaccinated with either the low-dose formulation (which was subsequently approved as GARDASIL®, Merck and Co., Inc., Whitehouse Station, NJ, USA) or placebo, 241 were enrolled in an extension of the study to obtain an additional 2 years of follow-up data for safety, efficacy, and immunogenicity. Eligible for participation in the extension were all subjects from Brazil and the Nordic countries who had participated in the dose-ranging study, had been vaccinated with either the low-dose vaccine or placebo formulations, had not discontinued during the initial 3-year study, and who agreed to participate. The recruited women in the US were not offered the possibility of enrolling in the extension phase as they were largely from universities and were finishing school, thus making this 2-year extension impractical for them.

**Figure 1:**
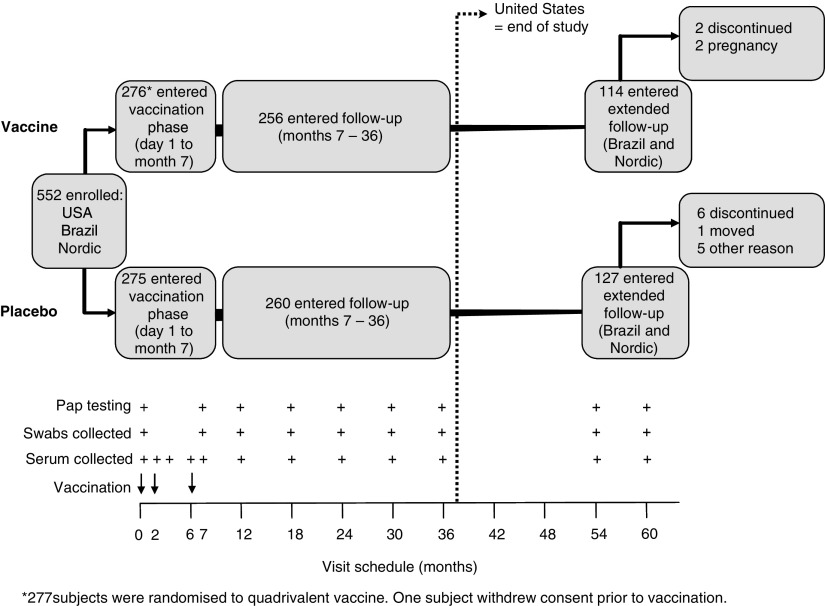
Trial design. Follow-up time for each woman in each study phase varies dependent on when she completed the last study visit. Discontinuations through month 36 can be found in Villa *et al* (2005).

This report presents the cumulative efficacy and immunogenicity results for all subjects who were randomised and vaccinated with low-dose quadrivalent HPV vaccine or placebo, through the extension period. Figure 1 shows the study design for the low-dose formulation and placebo, including the extended follow-up phase.

### Study vaccine

The quadrivalent vaccine consisted of a mixture of four recombinant HPV type-specific VLPs composed of the L1 major capsid proteins of HPV types 6, 11, 16, and 18 synthesised in *Saccharomyces cerevisiae* (Lowe *et al*, 1997; Koutsky *et al*, 2002; Ault *et al*, 2004). The low dose formulation is comprised of 20 *μ*g of HPV 6 VLP, 40 *μ*g of HPV 11 VLP, 40 *μ*g of HPV 16 VLP, and 20 *μ*g of HPV 18 VLP, formulated with 225 *μ*g of aluminium adjuvant in a total carrier volume of 0.5 ml. The four VLP types were purified and adsorbed onto amorphous aluminium hydroxyphosphate sulphate adjuvant (AAHS). The placebo contained the same adjuvant and was visually indistinguishable from vaccine.

### Clinical follow-up

During the initial 3-year study, a 0.5-ml dose of quadrivalent vaccine or placebo was administered by intramuscular injections at day 1, month 2, and month 6. A gynaecological examination was conducted at day 1 and at months 7, 12, 24, and 36. A ThinPrep™ Pap test (Cytyc, Boxborough MA, USA) and external genital, lateral vaginal, and cervical swabs for HPV DNA testing were obtained at day 1 and at months 7, 12, 18, 24, 30, and 36. External anogenital and vaginal lesions noted during the study were biopsied. Serum samples were obtained at day 1 and months 2, 3, 6, 7, 12, 18, 24, 30, and 36. In the extension, 241 subjects from Brazil and the Nordic countries underwent two further years of follow-up. Subjects enrolled in the extension phase had additional follow-up visits at months 54 and 60, which included gynaecologic examination, cervicovaginal sampling for HPV DNA, serum anti-HPV and Pap testing (Figure 1).

### Laboratory analyses

All Pap Testing and histologic evaluations were performed within the study. Pap tests were read using The Bethesda 2001 System (Solomon *et al*, 2002). Subjects referred for colposcopy had discrete areas of abnormality biopsied using separate instruments. All biopsies were processed independently to avoid HPV DNA contamination. Biopsies were first read for clinical management by pathologists at a central laboratory (Diagnostic Cytology Laboratories, Indianapolis, IN, USA) and then read for end point determination by a blinded panel of four pathologists (Villa *et al*, 2005). HPV 6, 11, 16, and/or 18 infection was defined using HPV DNA PCR and serology. Swabs, biopsy fragments and, later, tissue thin sections cut adjacent to sections used for histopathology were used for HPV DNA detection using HPV 6-, 11-, 16-, or 18-specific L1, E6, and E7 primers in HPV Multiplex Real-Time PCR assays (Villa *et al*, 2005). Serum anti-HPV 6, 11, 16, and 18 immunoglobulin levels were measured using a competitive Luminex immunoassay (cLIA), which were reported in arbitrary units (milli-Merck Units per millilitre or mMU/ml) relative to the standard curves generated for each individual HPV type (Opalka *et al*, 2003). An audit conducted by Merck Research Laboratories concluded that there was a deviation from the standard operating procedure (SOP) for testing a subset of serum samples from the protocol. Approximately, 0.1% of day 1 serology results and 0.4% of postvaccination serology results were determined to have been tested outside of the SOP. All day 1 serum samples that were tested out of compliance with the SOP were reanalysed. The remaining nonconformant test results were removed from the database.

### Primary case definition

The study's efficacy objective was to evaluate the impact of the quadrivalent HPV L1 VLP vaccine with respect to the composite end point of persistent HPV 6-, 11-, 16-, or 18-related persistent infection or cervical or external anogenital or vaginal disease, as compared to placebo. The persistent infection component of the primary efficacy end point was defined as follows: the same vaccine-HPV-type DNA detected in cervicovaginal samples collected at two or more consecutive visits (required to be at least 4 months apart unless at least one sample was tissue diagnosed as cervical, vaginal, or vulvar disease by the Pathology Panel) or vaccine-HPV-type DNA detected in a sample collected during the last visit before being lost-to-follow-up. Vaccine-HPV-type-related disease was defined as a tissue sample diagnosed by consensus of the Pathology Panel as cervical intraepithelial neoplasia (CIN), adenocarcinoma *in situ* (AIS), vulvar intraepithelial neoplasia (VIN), vaginal intraepithelial neoplasia (VaIN), external genital warts or cervical, vulvar, or vaginal cancer with vaccine-HPV-type DNA detected in tissue from, or a swab of, the same lesion and in cervicovaginal samples obtained at the visit antecedent to the biopsy visit. The latter condition was not required if vaccine-HPV-type DNA was detected in biopsy thin sections.

### Statistical analysis

The analyses presented here were conducted based on an interim frozen file created using a visit cutoff date of 13 February 2006. The interim frozen file was generated for an FDA Advisory Committee meeting. At the time of the interim frozen file, 226 out of 241 (94%) of subjects had complete data through month 60 that was available for analysis. The primary analysis of efficacy was conducted in type-specific perprotocol populations which consisted of subjects who were PCR and seronegative to HPV 6, 11, 16, or 18 at enrolment, remained PCR-negative to the same vaccine-HPV-type(s) (to which they were naïve at enrolment) through 1 month postdose three, received three doses of vaccine or placebo within 1 year, and did not violate the protocol. For example, to be eligible for the perprotocol population for HPV 16-related end points, a subject had to be both HPV 16 seronegative and HPV 16 DNA-negative by PCR at enrolment, and remain HPV 16 DNA-negative through 1 month postdose three. Prevalent or incident infection with HPV 6, 11, or 18 was not an exclusion criterion for HPV 16-related end points.

Follow-up for case ascertainment in the perprotocol population started 1 month postdose three. Vaccine efficacy (VE) was defined as 100% (1−(risk of becoming a case in the vaccine group/risk of becoming a case in the placebo group)). An exact conditional procedure was used to evaluate VE under the assumption that the numbers of cases in the vaccine and placebo groups are independent Poisson random variables. The number of cases in the vaccine group follows a binomial distribution, which is conditional on the total number of cases in the two vaccination groups combined being fixed. This conditional binomial distribution which accounts for any differential follow-up was used as the basis for calculating the point and exact 95% 2-sided confidence interval (CI) estimates of VE (Chan and Bohidar, 1998). Each subject's follow-up time was computed by calculating the number of person years between the specified starting time point and the date she became a case, or the date she underwent definitive therapy (cervical end points only) or her final visit date (for noncases). If a subject developed more than one end point, her date of becoming a case was the date at which the first end point was detected.

Supportive prespecified analyses were conducted in a modified-intention-to-treat (MITT) population that included all subjects who were naïve to the relevant HPV type(s) at enrolment and had received at least one vaccination. Protocol violators were included. Follow-up for case ascertainment in the modified-intention-to-treat population started 30 days after the first vaccination.

Anti-HPV 6, 11, 16, and 18 responses were measured in quadrivalent vaccine recipients who were seronegative to the relevant HPV type(s) at day 1 and remained PCR-negative to the same HPV type(s) through month 60, and who had serology data at month 60. Anti-HPV 6, 11, 16, and 18 responses were also measured in placebo recipients who were seropositive and PCR-negative to the relevant HPV type at day 1 and who had serology data at month 60.

### Role of the funding source

The studies were designed by the sponsor (Merck and Co., Inc.) in collaboration with clinical site investigators. The sponsor collected the data, monitored the conduct of the study, performed the statistical analysis and coordinated the writing of the manuscript with all authors. Data were unblinded for statistical analyses after the data were screened for accuracy and completeness, protocol violators were identified, and the databases were locked for the primary analysis at month 36 (Villa *et al*, 2005). In the extension, the subject and the investigator, study site personnel, laboratory personnel conducting the clinical assays, and the Pathology Panel used in end point adjudication remained blinded to vaccination group. The authors were actively involved in data analysis and interpretation, and approved the final manuscript. All authors vouch for the veracity and completeness of the data and the data analyses.

## RESULTS

A total of 552 women were randomised to receive either quadrivalent HPV vaccine or placebo (Figure 1). Among these subjects, 241 women from Brazil and the Nordic countries were enrolled in the extended follow-up study. Baseline characteristic were similar between treatment groups in both the overall cohort, and among subjects with extended follow-up (Table 1). As of the time of this report, 226 of 241 extension subjects completed the study through month 60. Two women in the quadrivalent vaccine group and six women in the placebo group discontinued the study before month 60 (Figure 1). Seven subjects had not completed the month 60 visit. Among those with a month 60 visit, the average time from the first vaccination was 5.03 years.

High sustained VE against persistent infection and disease was observed through 5 years postenrolment (Table 2). In the perprotocol efficacy analysis (all subjects through 3 years and extension subjects through 5 years), 45 of the total 47 cases of HPV 6-, 11-, 16-, or 18-related persistent infection were observed in the placebo cohort. Among quadrivalent vaccine recipients, there was one case of HPV 16 DNA detection at the last visit before loss to follow-up (month 36), and one case of verifiable persistent infection attributed to HPV 18 infection. For this subject, HPV 18 DNA was detected at months 12 and 18 only. Subsequent time points tested HPV 18 DNA-negative. Both of these subjects in the quadrivalent vaccine group were cases in the initial 3-year efficacy analysis (Villa *et al*, 2005). Therefore, during the extension phase, all new cases of persistent infection (11 total) were observed in the placebo cohort. Through 5 years, in the perprotocol population, there were no cases of HPV 6-, 11-, 16-, or 18-related CIN or external anogenital or vaginal lesions in vaccine recipients, and six cases in placebo recipients (efficacy=100%; 95% CI: 12.4–100%). Statistical significance was not reached for the disease end points separately. Thus, among quadrivalent vaccine recipients, there were no breakthrough cases of infection or disease during the extended follow-up period.

High efficacy through 5 years was also observed when considering only study data from those perprotocol subjects who were eventually enrolled in the extension phase (104 vaccine subjects *vs* 120 placebo subjects). Among the 224 extension subjects, there was one case of infection in quadrivalent vaccine recipients (HPV 18 DNA detection at months 12 and 18, *vide supra*) and 22 cases of persistent infection or disease in placebo recipients (efficacy=95.1%; 95% CI: 69.4–99.9%).

We also analysed VE in a MITT population that included subjects who were HPV 6, 11, 16, or 18 naïve at enrolment and that received at least one dose of vaccine or placebo, regardless of general protocol violations (Table 3). Such subjects approximate the population of adolescent and young adult women who received at least one dose of the vaccine before exposure to an HPV type targeted by the vaccine. The observed efficacy against infection was 93.5% (95% CI: 82.5–98.3%). No cervical, vulvar, or vaginal disease or genital warts due to HPV 6, 11, 16, or 18 was observed in quadrivalent vaccine recipients (efficacy=100%; 95% CI: 55.3–100%).

As there was an 18-month gap between month 36 (the last visit under the original protocol) and month 54 (the first visit in the extension phase) there was a small chance that cases of persistent infection may have occurred in this window which were not captured within the study. A sensitivity analysis was thus conducted to estimate the impact of the 18-month gap on VE. The sensitivity analysis involved modification of the definition of persistent infection such that an extension subject with a single time positive infection detected at month 36 or 54 without confirmation of persistence (including samples from any unscheduled biopsies between month 36 and month 54), was additionally counted as a case of persistent infection. The definition of the disease component did not change. Using this modified definition, four placebo and two vaccine recipients were conservatively counted as additional cases of persistent infection due to vaccine-type DNA detected at a single visit at either month 36 or 54, resulting in a total of four cases in the vaccine group and 50 cases in the placebo group. No subject who had an unscheduled biopsy performed between months 36 and 54 had vaccine-type DNA detected in that biopsy sample. No disease cases caused by vaccine HPV types were added in the sensitivity analysis. The VE estimate from the sensitivity analysis was 92% (95% CI: 78–98%).

Vaccine-induced anti-HPV GMTs were measured using a competitive assay, which measures the antibody response to known neutralising epitopes. This provides a specific measure of immunogenicity that is clinically related to protection against HPV infection. At month 7 (1 month after dose three) anti-HPV GMTs among quadrivalent vaccine recipients were substantially higher than GMTs among placebo-recipients with a previous history of natural HPV infection (Table 4). Though the number of placebo subjects who were infected before vaccination is too small to make meaningful comparisons, at month 60, vaccine-induced immune responses appear to remain at or above the GMTs observed in placebo-treated women who mounted an immune response presumably associated with clearance of HPV infection.

## DISCUSSION

The present study demonstrates sustained long-term efficacy and immunogenicity for a multivalent vaccine targeting the HPV types, which are responsible for a significant proportion of anogenital cancers and genital warts. Over 5 years of follow-up, the incidence of HPV 6-, 11-, 16-, or 18-related disease was reduced by 100% among women who received at least one dose (MITT population), compared with placebo recipients. A persistent and measurable immune response equal to or greater than that observed during natural infection was maintained through 5 years. Although the study was not originally powered to conduct analyses of VE for the disease end points, separately phase III studies conducted in >18 000 women have shown this quadrivalent vaccine to be 100% effective against cervical, vaginal, and vulvar precancerous lesions and genital warts through 2 years of follow-up in the perprotocol efficacy population (Sattler, for the FUTURE I Investigators, 2005; Skjeldestad, for the FUTURE II Steering Committee, 2005), providing encouraging information about the potential long-term efficacy of this vaccine in protecting against clinically relevant disease.

Importantly, there were no breakthrough cases of disease through the entire 5-year study. The design of the study allowed for the full ascertainment of HPV 6-, 11-, 16-, and 18-related genital disease. Both the external genitalia and cervicovaginal tract were sampled. Subjects underwent external genital and cervicovaginal examination at 6-month intervals through 36 months, and then at months 54 and 60. It should be noted that all subjects underwent a mandatory colposcopy at month 36, increasing the likelihood that cervical disease, if present, would be detected. Unscheduled biopsies during the 18-month gap were included in the analysis, and showed that no HPV 6-, 11-, 16-, or 18-related disease was observed among quadrivalent vaccine recipients. In addition, there were no breakthrough cases of infection during the extension phase. All of the 11 cases observed during the extension were in the placebo group (perprotocol analysis). These data support the high observed 5-year duration of efficacy.

The WHO defines persistent infection as detection of the same HPV DNA in cervicovaginal specimens obtained in follow-up visits 6–12 months apart in women who are naive for the relevant type at baseline (Pagliusi and Aguado, 2004). An additional strength of the study is the conservative definition of persistent infection, which included all cases where HPV DNA was detected at two consecutive visits required to be at least 4 months apart unless at least one sample was tissue diagnosed as cervical, vaginal or, vulvar disease by the Pathology Panel. In addition, subjects with HPV DNA detected at the last visit on record were conservatively counted as cases of persistent infection. Our sensitivity analysis included cases of single-time infection. VE remained high when including such cases. In the perprotocol population, when excluding cases due to HPV DNA detection at the last visit on record, only one subject in the quadrivalent vaccine group had vaccine-type DNA detected at two consecutive visits (months 12 and 18 only) through 5 years of follow-up.

A potential limitation of the present study is the relatively small number of women who were enrolled in the extension phase. In addition, the study did not follow American women through the extension period due to the predicted difficulties associated with maintaining enrolment for two additional years, as most were recruited from college campuses. However, a previous study of the monovalent HPV 16 component of the quadrivalent vaccine followed 2391 American women through 4 years (Mao *et al*, 2006). In this study, VE in the perprotocol population was 100% for preventing HPV 16-related cervical dysplasia of any grade severity, suggesting that VE is not affected by race or region, and should remain equally high in the US population. Another potential limitation of the current study is the 18-month gap that ensued between month 36 and month 54. A previous study has shown that the median time to clearance of persistent HPV 16 infection is 20.7 months (Mao *et al*, 2006). Thus, there was a potential for bias with respect to efficacy estimates for persistent infection, as HPV DNA detection at month 36 could be due to transient infection, contamination, or true persistent infection, which may have cleared by month 54. It should be noted that the observed VE against persistent infection (95.5%) is higher than that previously reported in the 3-year analysis published by Villa *et al* (2005) (89%). Three subjects in the primary 3-year analysis had HPV 16 DNA detected at the last visit on record (month 36). Two of these subjects had extended follow-up and a subsequent month 54 swab which was DNA-negative. Consequently, there was no observed persistence of infection, although the possibility that infection was present between months 36 and 54 cannot be ruled out. It has been established that histologic changes of the cervix become apparent within 1.5 years of initial HPV infection (Sattler, for the FUTURE I Investigators, 2005) and genital warts may develop even faster (Winer *et al*, 2005). Thus, even with the 18-month gap, the present study was not limited in its ability to detect clinically relevant HPV-related disease.

Ideally, a prophylactic HPV vaccine would be given to young adolescents before the initiation of sexual activity since HPV is transmitted through genital contact. Our primary analysis considered a susceptible population of women who were naïve to vaccine-HPV-types at baseline. All sexually active individuals are at risk for HPV infection and disease, including those who do not engage in penetrative sexual intercourse. A recent study has shown the 24-month cumulative incidence of HPV infection among virgins before initiation of penetrative sexual intercourse was 15.3% (Winer *et al*, 2003). The fact that men and women remain at risk of HPV infection as long as they are sexually active, in combination with the lack of effective means to prevent HPV infection in sexually active people, requires vaccine-induced protection to be long-lived. Recently, a bivalent HPV 16/18 vaccine showed high efficacy against persistent HPV 16 or 18 infections through 4.5 years (Harper *et al*, 2006). In the present study, we have shown that a quadrivalent HPV vaccine formulated on proprietary aluminium adjuvant to be highly efficacious and immunogenic through 5 years. Vaccine-induced responses at month 60 remained at or above the response level seen by natural infection. The vaccine-induced immune response appears highest for HPV 16, however, as the monoclonal antibodies used in the competitive assays each recognise type-specific epitopes, one cannot draw conclusions with regard to the relative immunogenicity of the four VLP components in the vaccine. The scale of the competitive immune response is dependent upon the particular attributes of each monoclonal antibodies, the epitope that they recognise, and their relationship to the standard curve generated for each individual HPV type tested. Other studies have shown that quadrivalent vaccine-induced responses in 10- to 15-year-old girls are substantially higher than the vaccine-induced responses observed in 16- to 23-year-old women (Block *et al*, 2006). Thus, it is likely that administration of quadrivalent HPV vaccine to female adolescents before sexual debut will induce long-term protective efficacy. Sexually active young women with few lifetime sex partners, similar to women enrolled in this trial, will also benefit from vaccination.

This study has demonstrated that a prophylactic quadrivalent HPV vaccine induced high-titre anti-HPV serum antibody levels and was highly effective through 5 years for prevention of persistent infection, neoplasia, and genital warts caused by HPV 6, 11, 16, and 18. This duration supports vaccination of adolescents and young adults, which is expected to greatly reduce the burden of cervical and other anogenital cancers, low- and high-grade intraepithelial neoplasias and genital warts.

## Figures and Tables

**Table 1:** Summary of subject characteristics by vaccination group at enrolment

	**Overall cohort**		**Subjects enroled in protocol extension**	
	**Vaccine (*n*=277)**	**Placebo (*n*=275)**	**Vaccine (*n*=114)**	**Placebo (*n*=127)**
Mean age (years)	20.2	20.0	20.5	20.3
Range (years)	16–23	13^a^–23	16–23	16–23
				
*Ethnic origin*				
Asian	7 (2.5%)	11 (4.0%)	0 (0%)	1 (0.8%)
Black	25 (9.0%)	18 (6.5%)	14 (12.3%)	15 (11.8%)
Hispanic	14 (5.1%)	20 (7.3%)	3 (2.6%)	3 (2.4%)
White	216 (78.0%)	214 (77.8%)	88 (77.2%)	99 (78%)
Other	15 (5.4%)	12 (4.4%)	9 (7.9%)	9 (7%)
				
*Region*				
USA	125 (45.1%)	126 (45.8%)	NA	NA
Brazil	94 (34.0%)	93 (33.8%)	72 (63.2%)	79 (62.2%)
Nordic	58 (20.9%)	56 (20.4%)	42 (36.8%)	48 (37.8%)

a One subject was 13 years and one subject was 15 years at the time of enrolment.

**Table 2:** Analysis of efficacy of the quadrivalent HPV vaccine against HPV 6-, 11-, 16-, or 18-related persistent infection or disease in the perprotocol population through 5 years postenrolment (all subjects through 3 years and extension subjects through 5 years)

	**Vaccine**				**Placebo**					
**End point^a^**	***n***	**Cases**	**Women-years at risk**	**Rate^b^**	***n***	**Cases**	**Women-years at risk**	**Rate^b^**	**Efficacy (%)**	**95% CI (%)**
*Infection or disease*	**235**	**2**	**767.9**	**0.3**	**233**	**46**	**747.4**	**6.2**	**95.8**	(**83.8, 99.5)**
Infection	235	2	764.7	0.3	233	45	749.7	6.0	95.6	(83.3, 99.5)
Disease	235	0	771.9	0.0	233	6	796.4	0.8	100.0	(12.4, 100.0)
CIN 1-3	235	0	755.5	0.0	233	3	774.9	0.4	100.0	(<0.0, 100.0)
Condyloma	235	0	771.1	0.0	233	3	796.2	0.4	100.0	(<0.0, 100.0)
										
*End points by HPV type*										
HPV 6-related	214	0	700.7	0.0	209	17	702.7	2.4	100.0	(75.7, 100.0)
HPV 11-related	214	0	700.7	0.0	209	3	712.4	0.4	100.0	(<0.0, 100.0)
HPV 16-related^c^	199	1	663.3	0.2	198	28	637.5	4.4	96.6	(79.2, 99.9)
HPV 18-related^c^	224	1	732.7	0.1	224	11	753.9	1.5	90.6	(35.6, 99.8)

*n*=no. of subjects included in the perprotocol population who had at least one follow-up visit.

a A subject appears only once within each row. A subject may appear in more than one row.

b Cases per 100 woman years at risk.

c Among quadrivalent vaccine recipients, there was one case of HPV 16 DNA detection before loss to follow-up (month 36), and a single case of verifiable persistent infection attributed to HPV 18 infection. For this subject, HPV 18 DNA was detected at months 12 and 18 only. The bold values are the data for the primary composite end point for which the study was designed and powered.

**Table 3:** Analysis of efficacy of the quadrivalent HPV vaccine against HPV 6-, 11-, 16-, or 18-related persistent infection or disease in the modified intention-to-treat population through 5 years postenrolment (all subjects through 3 years and extension subjects through 5 years)

	**Vaccine**				**Placebo**					
**End point^a^**	***n***	**Cases**	**Women-years at risk**	**Rate^b^**	***n***	**Cases**	**Women-years at risk**	**Rate^b^**	**Efficacy (%)**	**95% CI (%)**
*Infection or disease*	**266**	**4**	**945.0**	**0.4**	**263**	**59**	**879.5**	**6.7**	**93.7**	(**83.0, 98.3)**
Infection	256	4	939.0	0.4	254	58	880.0	6.6	93.5	(82.5, 98.3)
Disease	266	0	951.9	0.0	263	10	953.3	1.0	100.0	(55.3, 100.0)
CIN 1-3	258	0	930.6	0.0	256	7	928.5	0.8	100.0	(30.8, 100.0)
Condyloma	265	0	950.4	0.0	261	4	955.0	0.4	100.0	(<0.0, 100.0)
										
*End points by HPV type*										
HPV 6-related	242	0	860.7	0.0	242	22	854.9	2.6	100.0	(81.9, 100.0)
HPV 11-related	242	0	860.7	0.0	242	4	868.9	0.5	100.0	(<0.0, 100.0)
HPV 16-related^c^	225	3	819.6	0.4	229	34	779.0	4.4	91.6	(73.3, 98.4)
HPV 18-related^c^	253	1	897.8	0.1	253	12	904.2	1.3	91.6	(43.3, 99.8)

*n*=no. of subjects included in the modified-intention-to-treat population who had at least one follow-up visit.

a A subject appears only once within each row. A subject may appear in more than one row.

b Cases per 100 woman years at risk.

c Description of cases among quadrivalent vaccine recipients: one case of confirmed persistent infection with HPV 18 DNA detected at months 12 and 18 only (same subject as perprotocol analysis); one case of confirmed persistent infection with HPV 16 DNA detected at months 7, 12, and 18; and two cases of HPV 16 DNA detected at the last visit on record (months 2 and 36 (same subject as perprotocol analysis)). The bold values are the data for the primary composite end point for which the study was designed and powered.

**Table 4:** Anti-HPV 6, 11, 16, and 18 geometric mean titres through 5 years (extension subjects only)

	**Vaccine recipients who were baseline seronegative and PCR-negative for a specific HPV type^a^**			**Placebo recipients who were baseline seropositive and PCR-negative for a specific HPV type**		
	***n***	**GMT (mMU/ml)**	**95% CI**	***n***	**GMT (mMU/ml)**	**95% CI**
*Month 7*						
HPV-6	77	559.7	(466.5, 671.5)	9	31.3	(14.9, 65.7)
HPV-11	83	642.4	(530.3, 778.2)	2	342.7	N/A
HPV-16	78	3889.1	(3147.1, 4806.1)	9	42.0	(13.8, 128.3)
HPV-18	82	755.5	(582.3, 980.1)	7	36.4	(12.3, 107.5)
						
*Month 36*						
HPV-6	77	88.1	(70.5, 110.0)	9	28.9	(14.5, 57.6)
HPV-11	79	78.0	(61.5, 99.0)	2	219.2	N/A
HPV-16	78	441.3	(350.3, 556.1)	9	21.5	(<12, 64.3)
HPV-18	82	50.5	(36.7, 69.5)	7	24.0	(9.9, 58.0)
						
*Month 60*						
HPV-6	77	66.5	(52.3, 84.6)	9	30.5	(14.9, 62.5)
HPV-11	83	67.6	(51.1, 89.3)	2	150.4	N/A
HPV-16	78	395.4	(303.2, 515.7)	8	16.0	(<12, 52.2)
HPV-18	82	43.7	(30.8, 62.1)	7	32.7	(9.3, 115.0)

a Subjects in the perprotocol population who were PCR-negative and seronegative to the relevant HPV type at day 1 and remained PCR-negative to the same HPV type through month 60.
